# Treatment of CD20-directed Chimeric Antigen Receptor-modified T cells in patients with relapsed or refractory B-cell non-Hodgkin lymphoma: an early phase IIa trial report

**DOI:** 10.1038/sigtrans.2016.2

**Published:** 2016-03-11

**Authors:** Wen-ying Zhang, Yao Wang, Ye-lei Guo, Han-ren Dai, Qing-ming Yang, Ya-jing Zhang, Yan Zhang, Mei-xia Chen, Chun-meng Wang, Kai-chao Feng, Su-xia Li, Yang Liu, Feng-xia Shi, Can Luo, Wei-dong Han

**Affiliations:** 1 Biotherapeutic Department, Chinese PLA General Hospital, Beijing, China; 2 Department of Immunology, Institute of Basic Medicine, School of Life Sciences, Chinese PLA General Hospital, Beijing, China; 3 Department of Geriatric Hematology, Chinese PLA General Hospital, Beijing, China

## Abstract

Patients with relapsed or refractory non-Hodgkin lymphoma have a dismal prognosis. Chimeric Antigen Receptor (CAR)-modified T cells (CART cells) that targeted CD20 were effective in a phase I clinical trial for patients with advanced B-cell lymphomas. We performed a phase IIa trial to further assess the safety and efficacy of administering autologous anti-CD20 CART (CART-20) cells to patients with refractory or relapsed CD20^+^ B-cell lymphoma. Eleven patients were enrolled, and seven patients underwent cytoreductive chemotherapy to debulk the tumors and deplete the lymphocytes before receiving T-cell infusions. The overall objective response rate was 9 of 11 (81.8%), with 6 complete remissions (CRs) and 3 partial remissions; no severe toxicity was observed. The median progression-free survival lasted for >6 months, and 1 patient had a 27-month continuous CR. A significant inverse correlation between the levels of the *CAR* gene and disease recurrence or progression was observed. Clinically, the lesions in special sites, specifically the spleen and testicle, were refractory to CART-20 treatment. Collectively, these results together with our data from phase I strongly demonstrated the feasibility and efficacy of CART-20 treatment in lymphomas and suggest large-scale patient recruitment in a future study. This study was registered at www.clinicaltrials.org as NCT01735604.

## Introduction

Non-Hodgkin lymphoma (NHL) is a hematological malignancy with high mortality and a poor prognosis. The expected 5-year and 10-year overall survival rates for subjects treated with standard chemotherapy are 58% and 43.5%, respectively.^1,2^ However, for relapsed and refractory NHL, the response rates to conventional salvage chemotherapy are approximately 40–50%. Patients previously treated with rituximab had a significantly worse progression-free survival (PFS) rate than patients who were rituximab-naive (29% vs 44%, respectively).^3–8^ In diffuse large B-cell lymphoma (DLBCL), an autologous hematopoietic stem cell transplant has become the standard of care for patients in their first relapse. However, the treatment-related mortality with allogeneic transplantation can reach up to 25%,^9^ and the fatalities from the autologous hematopoietic stem cell transplant procedure are even higher.^10^ Therefore, the search for novel therapeutic modalities that will yield improved and sustained outcomes in such patients is continuing.

Adoptive cell transfer, typically represented by tumor-specific Chimeric Antigen Receptor-modified T (CART) cells, holds great promise as a tumor therapy.^11,12^ The CD20 antigen on the surface of B-NHL cells is a well-established immunotherapy target for lymphoma. For indolent B-cell and mantle cell lymphomas, the efficacy and safety of CART-20 has been confirmed.^13^ However, for aggressive forms of lymphoma, such as DLBCL, there have been no relevant studies. Kochenderfer *et al.*^14^ reported on two patients with high-risk DLBCL for whom complete remission (CR) was achieved after treatments with CART-19 cells, but the pathological subtypes of the lymphomas in both subjects were not-otherwise specified, which has a better prognosis than the activated-B-like-cell subtype. We previously reported on the efficacy of the use of CART-20 cells to treat patients with refractory advanced DLBCL. Overall, five of six patients were evaluated for clinical efficacy; of these, one of the two patients with no bulky tumors had a 14-month sustained CR following the cell infusion as the sole treatment. However, selected delayed toxicities of CART-20 treatment were observed, including a massive hemorrhage of the alimentary tract and an aggressive intrapulmonary inflammation surrounding the extranodal lesions.^15^

Based on the results from our prior study, we revised the eligibility criteria for the patients in the Phase IIa study to reduce toxicity and evaluated the efficacy and *in vivo* persistence of CART-20 cells in subjects with high-risk relapsed or refractory B-cell NHL. In this report, we enrolled 11 patients with relapsed or chemotherapy refractory B-cell NHL, including 1 with a previous autologous hematopoietic stem cell transplant treatment and 1 with a primary cutaneous B-cell lymphoma. In combination with the previous results of phase I clinical trial, our study provides further support for the use of CART-20 as a clinical treatment for patients with NHL and raises the possibility of using CART-20 in an early disease stage.

## Materials and methods

### Study design

This single institution, open-label, Phase IIa escalation study (ClinicalTrials.gov identifier: NCT01735604) was performed in the Department of Bio-therapeutics of the Chinese PLA General Hospital. The study protocol was approved by the ethics committee of the Chinese PLA General Hospital. All patients provided informed consent upon enrollment in accordance with the Declaration of Helsinki Principles. No commercial sponsor was involved in the study.

**Figure 1:**
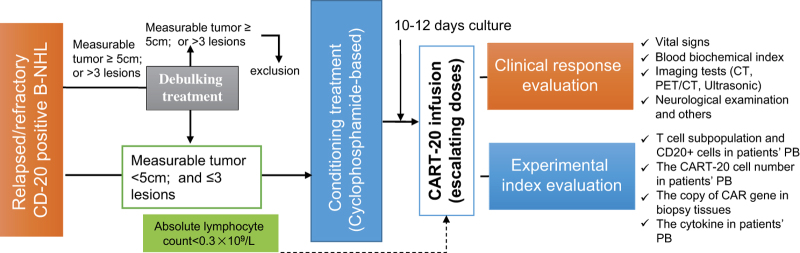
Clinical protocol design. Patients with tumors that had a diameter <5 cm or who had ⩽3 lesions provided samples of peripheral blood mononuclear cells from which CART cells were prepared 10–12 days before infusion. Within this time, some patients were given lymphocyte-depleting chemotherapy as described. The infusion was given using a split-dose approach over 4–5 days. Endpoint assays were conducted on study weeks 4–6. CART cells, Chimeric Antigen Receptor-modified T cells; PET-CT, positron emission tomography-computed tomography.

The patients underwent cytoreductive chemotherapy for tumor debulking and lymphocyte depletion between days −7 and −3 before T-cell infusion. However, according to the judgment of physicians, if patients had a small tumor burden (maximum diameter <5 cm or number of lesions ⩽3) and a lymphocyte deficiency (absolute lymphocyte <0.3×10^9^ l^−1^, regardless of the presence of regulatory T cells, T lymphocytes or B lymphocytes). Taking into account the needs of reducing lymphocytes, excluding the interference of pre-condition and minimizing the damages to patient’s bone marrow and immune system, we selected the shortest chemotherapeutic regimens include Cyclophosphamide that were capable of inducing a reaction of tumor in the short term as pre-condition regimen in this trail (Table 1). The patients received escalating doses of CART-20 cells split into 3–5 doses on consecutive days beginning on day 0 (Figure 1).

Therapy was terminated if uncontrolled toxicity persisted or recurred; otherwise, treatment was continued until progression of lymphoma after infusion, the patient’s request or the investigator’s judgment. The primary study endpoints were safety and tolerability in all patients. The secondary endpoints included PFS.

### Patients

All 11 patients had histologically confirmed relapsed or refractory CD20^+^ NHL and were enrolled in this study between May 2014 and June 2015. The clinical data set cutoff timepoint is at the end of November of 2015. We defined refractory lymphoma as progression or incomplete remission at 4 weeks after the end of the most recent chemotherapy or anti-CD20 Ab therapy. Other inclusion criteria were: age ⩾18 years, Eastern Cooperative Oncology Group (ECOG) performance status 0–3, life expectancy of at least 3 months, adequate hematological and renal function (absolute neutrophil count ⩾1.5×10^9^ l^−1^, platelet count ⩾100×10^9^ l^−1^, bilirubin ⩽1.5×the upper limit of the normal range). Treatment with prior cytotoxic or biologic therapies must have ended at least 4 weeks previously. Patients were excluded if they had evidence of involvement of the lung or alimentary canal, uncontrolled bulky lymphoma (maximum diameter ⩾5 cm or number of lesions >3) after debulking treatments, an uncontrolled infection requiring antibiotics, class III or IV cardiac disease or other specified cardiac conditions, or uncontrolled diabetes.

### Generation and expansion of CART-20 cells

The constructs of CAR20, the expansion, culture and the verification of transduction efficiency of CART-20 cells were primarily performed as described previously in our clinical trial.^15^ Briefly, peripheral blood mononuclear cells were activated with immobilized CD3 antibodies (Takara, Toyko, Japan) and recombinant interleukin-2 (500 U ml^−1^; PeproTech, Rocky Hill, NJ, USA). Lentivirus-mediated *CAR* transduction was performed on day 3 of the cell culture. After transduction, T-cell lines were expanded in the presence of interleukin-2 (500 U ml^−1^). The composition and purity were assessed by fluorescence-activated cell sorting, and the cells were harvested beginning on days 10–12.

### Response criteria, staging and follow-up

Clinical responses were assessed according to the recommendations of the International Workshop NHL Response Criteria.^16^ The toxicity and adverse events were graded using the National Cancer Institute Common Terminology Criteria of Adverse Events version 3.0 (http://ctep.cancer.gov/). The end of the follow-up period was 1 November 2015, and the median follow-up period was 8 months. Disease staging using computed tomography (CT) and whole-body 2-[18F]-fluoro-2-deoxy-D-glucose positron emission tomography (FDG-PET) scans was performed at the time of study entry. The CT was repeated during follow-up every 2 months (first year), every 6 months (years 2–3) and annually thereafter. An FDG-PET study was performed at 4–6 weeks after treatment in all patients. Extensive laboratory tests, including the evaluation of lymphocyte subpopulations, and a bone marrow biopsy were performed at baseline and repeated at regular intervals during the study period and during follow-up.

### Statistical analysis

The Kaplan–Meier method was used to calculate PFS. PFS was calculated from the first infusion of CART-20 cells until relapse or disease progression. Transgene expression and multiplex analyses were summarized over time using descriptive statistics. The univariate Cox-PH-regression model was used to estimate the hazard ratios and their 95% confidence interval. The data were plotted using GraphPad Prism version 5.0 (San Diego, CA, USA). Two-way analysis of variance was used to determine the significance of the differences between the means in all experiments. The significance between groups was determined using a *t*-test or Fisher’s exact test. A *P*-value<0.05 was considered to be statistically significant.

## Results

### Overall clinical responses

**Figure 2:**
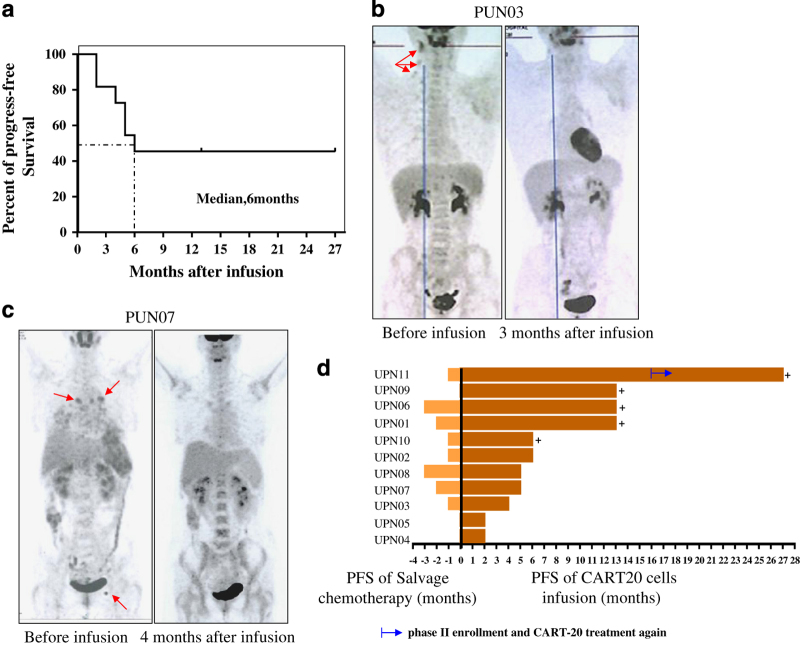
Clinical responses of all patients. (**a**) The progression-free survival (PFS) for all participating patients. More than 50% of the patients achieved a 6-month PFS. (**b**, **c**) The positron emission tomography-computed tomography (PET-CT) images of patients UPN03 and UPN07, respectively. The higher uptake in the cervical lymph node of UPN03 disappeared at the 3-month time point after the Chimeric Antigen Receptor-modified T-20 (CART-20) cell infusion. The lesions of patient UPN07 at the mediastinum and groin disappeared at the 6-month time point after the CART-20 cell infusion. (**d**) The time of progression-free survival after the CART-20 cell infusions was significantly longer than that observed after the last salvage chemotherapies. The light brown bars denote responses after salvage chemotherapy; the dark brown bars demonstrate the PFS after the CART-20 cell infusion.

All patients were assessed for responses 4–6 weeks after the CART-20 cell infusion. The therapeutic outcomes are summarized in Table 1. Seven patients received cytoreductive chemotherapy, including cyclophosphamide for debulking and lymphocyte depletion, before the infusion, whereas the other four patients were not treated on the basis of a smaller tumor burden and a lower level of lymphocytes. The overall response rate was 81.8%, with 54.5% of the patients (6/11) achieving a CR and 27.3% (3/11) achieving partial remissions (PRs). The other two patients had stable disease. As shown in Figure 2a, the median PFS was 6 months, with a median follow-up of 8 months (range: 5–27 months). Of the eight patients with DLBCL, four achieved CR, three achieved PR and one had stable disease after infusion of CART-20 cells with no further treatment. Two of the three patients with indolent B-cell malignancies achieved CR and one had stable disease.

Prior to CART-20 infusions, eight patients had received at least six cycles of chemotherapy, and five of them (UPN02, -03, -04, -05 and -07) relapsed and developed advanced lymphoma. CART-20 infusion led to CR for two patients (UPN03, -07), and two patients (UPN02, -04) achieved PR. All three patients (UPN08, -09, and -10) with primary refractory lymphoma achieved remission after infusion, and two of them achieved CR. Patient UPN03, the only patient in this study who had received a previous autologous stem cell transplantation achieved a longer CR, 4 months, after CART-20 cell infusion compared with 1-month tumor regression after autologous stem cell transplantation treatment (Figure 2b). Patient UPN07, with indolent lymphoma, had advanced disease after receiving six prior regimens before enrollment in our study. After treatment with the CART-20 protocol, this patient achieved a CR that was ongoing for 5 months after infusion (Figure 2c).

Advanced age of lymphoma patients is associated with an increase in the mortality following chemotherapy. In our clinical trial, there were four patients over 60 years of age. Patient UPN10, a 64-year-old man, had DLBCL activated-B-like-cell with stage II bulk lesions and had just achieved PR after two cycles of R-CHOP. Before being enrolled in our study, he had 3-cm diameter residual lesions in the submaxillary nodes. After an adjusted CHOP regimen given as conditioning treatment, he received the infusion of CART-20 cells. At the fourth week after infusion, he achieved CR that lasted for more than 6 months. Patient UPN11, a 70-year-old man with refractory DLBCL germinal center b-cell origin, achieved CR after the first infusion of CART-20 in our phase-I trial. At the sixteenth month, we observed increases in the numbers of CD20^+^ B cells accompanied by a decrease in the level of CART-20 gene in peripheral blood (PB) of this patient. Furthermore, at this late time after CART-20 cell infusion, the patient did not exhibit a deficiency in immunoglobulins. Based on the safety of CART-20 and the reluctance of the patient to undergo chemotherapy, we administered thesecond CART-20 infusion to him to prevent the recurrence of lymphoma. Consistent with our expectations, he had a prolonged CR accompanied with a decrease of B cells that continued for >27 months, which was the longest remission observed in our study (Supplementary Appendix Figure 1).

Five patients (5/11: UPN02, -03, -04, -07 and -08) relapsed after achieving remissions of 2–6 months after CART-20 infusion. Although beyond our study timeline, to our knowledge, patient UPN04 was still in remission induced by radiotherapy after relapsing following the infusion. Overall, all patients achieved clinical benefits in the PFS in our study compared with their previous treatments (Figure 2d).

### Characterization and dose of CART-20 cells

After 10–12 days of culture, the cells were released for infusion. A mean of 95±12.7% of the infused cells were CD3^+^ cells, principally composed of the CD8^+^ subset (average 76%). A median of 30.5% (range 21.2–41%) of the T cells were positive for *CD20-CAR* expression, as shown by flow cytometry. The final number of cells infused into each patient is summarized in Supplementary Table 1. The prominent cytolytic activities of the CART-20 cells were previously described by our team. We found that some of the CD3^+^ T cells expressed markers of the central memory (CD45RO^+^/CD62L^+^, range: 45.32–99.03%) and effector memory phenotypes (CD45RO^+^/CCR7^−^/CD62L^−^, range: 0.97–83.72%). The ratios of these central memory and effector memory cells in the total infused T cells are summarized in Supplementary Table 2.

### The long-term persistence of CART-20 cells

**Figure 3:**
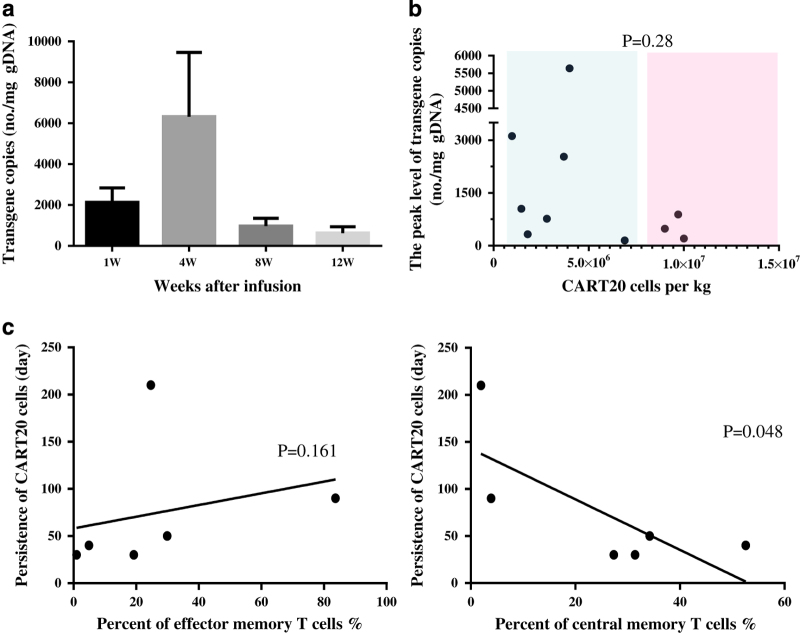
Characteristics and influential factors of expansion of the Chimeric Antigen Receptor-modified T-20 (CART-20) cells *in vivo*. (**a**) The time course shows that the peak levels of the CART-20 cells detected by qRT-PCR were observed at approximately 4 weeks after infusion and gradually decreased thereafter. (**b**) Detection of CART-20 cells at 1 week and 4 weeks after infusion was independent of the infused T-cell dose. (**c**) A copy number less than 200 (no./mg gDNA) was regarded as CART-20 cell negative. The last observance of positive CART-20 cells was independent of the absolute effects of memory and central memory T cells.

T-cell persistence is an important factor for successful tumor eradication. In our study, the persistence of CART-20 cells was assessed *in vivo* by means of a quantitative real-time PCR (qRT-PCR) assay of the PB mononuclear cells, the detection method was carried out as described previously study.^15^ The peak levels of CART-20 cells (range: 800–255,044 copies of CART cells per microgram of DNA) measured by qRT-PCR were observed at ~4 weeks (range: 22–35 days) after infusion. Although they gradually declined after 4 weeks, the *CAR* genes in PB of most of the patients (except patient UPN04) were still detected by qRT-PCR at a high level (200 copies of CART cells per μg of DNA) at the 12th week after infusion (Figure 3a). As of 1 November 2015, the *CAR* gene was detected by qRT-PCR for more 2 years after the infusion in patient UPN11, who had the longest remission (27 months; Supplementary Appendix Figure 1).

To avoid different responses of patients caused by variable doses of the CART-20 cells (range: 0.41×10^7^–1.46×10^7^ kg^–1^), we studied the relationship between the copy number and the infused T-cell dose. The doses of cells were determined according to the total amount of cells available after production. Within the range of 0.41×10^7^–1.46×10^7^ kg^–1^, the dose of infused T cells did not correlate with the peak levels of the copy number of CART-20 cells (*P*=0.28; Figure 3b).

The choice of the T-cell subset to utilize is one of the important aspects of this therapy. The percentages of effector memory T cells (CD3^+^/CD45RO^+^) and central memory T cells (CD3^+^/CD45RO^+^/ CCR7^+^/CD62L^+^) in the total of infused CART-20 cells were assessed by flow cytometry. However, we did not find a positive correlation between the time for which the CART-20 cells persisted (where negative was defined as <200 copies per microgram of genomic DNA) and the relative composition of effective memory T cells and central memory T cells (*P*=0.161 and *P*=0.148, respectively; Figure 3c).

### *CAR* molecule levels and the relapse of lymphoma have a reverse correlation with CD20^+^ target cell numbers

**Figure 4:**
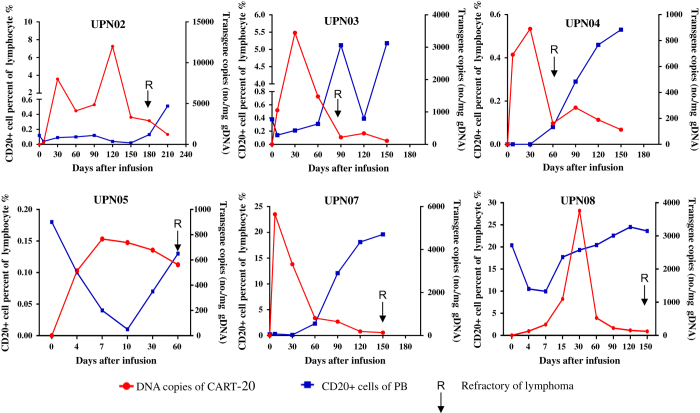
The numbers of CD20^+^ lymphocyte cells by flow cytometry and the copy numbers of Chimeric Antigen Receptor (*CAR*) molecules by quantitative real-time-PCR in the peripheral blood of relapsed patients after Chimeric Antigen Receptor-modified T-20 (CART-20) cell infusion. Relapses occurred after the numbers of copies dropped and the numbers of B cells increased. An ↓ indicates the time of relapse.

CD20^+^ cells and copy numbers of *CAR* molecules in patients’ PB samples were serially measured post CART-20 infusion by flow cytometry and qRT-PCR, respectively. After infusion of CART-20 cells, B-cell aplasia occurred in all patients and was associated with an increase in *CART-20* copy numbers (Supplementary Appendix Figure 2). Five patients who subsequently had remission had a relapse between 60 and 180 days after infusion of CART-20 cells. We found that they all had a relapse of the CD20-positive lymphoma following the recovery of the polyclonal B cells from aplasia and the decline of copies of *CAR* gene in PB when the lymphoma advanced (Figure 4).

### CART-20 cell traffic to tumor sites

**Figure 5:**
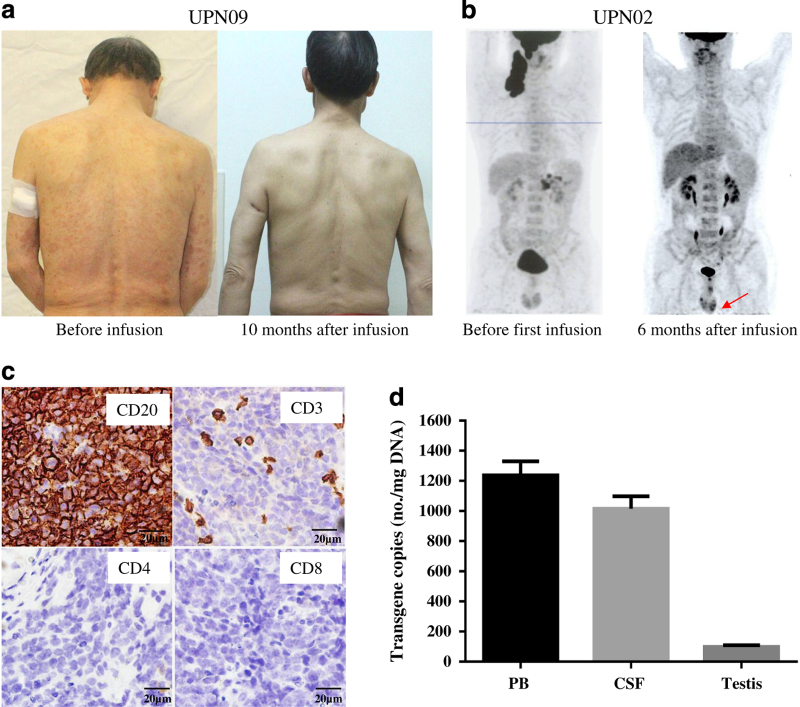
Responses of the special lesions to Chimeric Antigen Receptor-modified T-20 (CART-20) cells. (**a**) The skin lesions of patient UPN09 completely disappeared, and the disappearance lasted up to 10 months or more, despite continued splenomegaly. (**b**) The new lesions in the testes of patient UPN02 emerged at 6 months after infusion. (**c**) Immunohistochemical examination of a needle biopsy of lesions in the testis from patient UPN02 at 6 months after CART-20 cell infusion showed that the tumor cells were CD20^+^, CD3^−^, CD4^−^, and CD8^−^. (**d**) The CART-20 cells in the peripheral blood, cerebral spinal fluid (CSF) and the testis of patient UPN02 were measured by quantitative real-time-PCR at 6 months after CART-20 cell infusion. PB, peripheral blood cells.

Good T-cell trafficking to the tumor site is a prerequisite for a treatment response, as is the reverse situation. Patient UPN09 had an advanced refractory marginal zone lymphoma. In addition to an enlarged spleen, he had an extranodal form of lymphoma that involved recurrent erythematous patch-like lesions of the skin and 13% lymphoma cells in the bone marrow before enrolling in our study. This subject had previously received five cycles of combination chemotherapy. One month after the administration of the CART-20 infusion, the skin lesions disappeared (Figure 5a) and the marrow lymphoma cells were not found by flow cytometry. At this time, the qRT-PCR analysis indicated high levels of CART-20 cells in the skin tissue (635 copies per microgram of genomic DNA) and bone marrow (455 copies per microgram of genomic DNA). The cutaneous lymphoma did not recur until the manuscript submitted. However, the size of the spleen did not become normal until at the third month after infusion. Due to concerns regarding bleeding from the spleen, we did not perform a biopsy to evaluate the homing of CART-20 cells to the spleen. After radiation therapy of the spleen, this patient achieved a CR for more than 10 months, which was at the end of the submission date.

Patient UPN02, whose primary lymphoma involved the facial muscles, jaw bone and neck lymph nodes before enrollment, obtained a continued PR for 6 months with our treatments. Unfortunately, new bilateral lesions in his testicles were discovered with a PET-CT scan at 6 months after infusion (Figure 5b). A testicular tumor was assessed with a fine-needle aspiration biopsy at 27 weeks after the CART-20 infusion. In this tumor, a large number of CD20^+^ cells were still present, and only a small number of CD3^+^, CD4^+^ and CD8^+^ T cells were present by immunohistochemistry (Figure 5c). The qRT-PCR analysis showed a low level of the *CAR* molecules in the testis, even though stronger qRT-PCR signals from the CART-20 cells were detected in the peripheral blood sample and cerebral spinal fluid at the same time (Figure 5d). These results indicated that the CART-20 cells did not effectively traffic to the tumor sites of the testis. Node biopsies were not performed in any of the other patients because of personal reasons or disease condition.

### Toxicity

The treatment regimen was generally well-tolerated; no grade four toxicities and no evidence of cytokine release syndrome were observed. Grade 1–3 adverse events, which were probably related to the infusion, are summarized in Table 2. Within half to 1 h after infusion, all patients had mild rigors and transient fever not higher than 40 °C and not longer than 4 h. During the follow-up days, none patient had fever again, even at the time when cytokines elevated. Elevations in alanine aminotransferase, aspartate aminotransferase and lactate dehydrogenase, hyperuricemia, hypoalbuminemia and hypokalemia were detected within 1 week after infusion, and these biochemical changes were reversible. UPN02 had a transient grade 3 hypokalemia for 1 day after suffering profuse sweating and anorexia resulting from fever following the infusion with the CART-20 cells. We evaluated patient serum samples from serial post-treatment time points and then analyzed the changes in the serum levels of cytokines correlated with the CART-20 treatment. The detection method was carried out as described previously study.^15^The cytokine levels, including those of tumor necrosis factor-α, interleukin-6 and CRP, were mildly elevated in all patients after the infusion; however, this did not constitute cytokine release syndrome (Supplementary Appendix Figure 3).

Patient UPN02 demonstrated an asymptomatic exudative inflammation of the lung at ~60 days after infusion without a significant elevation in the cytokines. Only a ground-glass-like change of the lower lung lobes detected with a CT scan and mildly elevated levels of interleukin-6 and tumor necrosis factor-α were observed. He was given dexamethasone by inhalation for 1 week. After the treatment, a repeat CT scan did not show the ground-glass changes.

Most patients were administered intravenous immune globulin monthly to prevent hypogammaglobulinemia. However, patient UPN09 declined the gammaglobulin infusion for 6 months, and then suffered a grade 3 herpes zoster infection at 238 days after CART-20 infusion in the presence of hypogammaglobulinemia. The cytopenias, including neutropenia, thrombocytopenia and anemia, related to cytoreductive chemotherapy are not listed.

## Discussion

CD20 is an ideal target antigen for NHL immunotherapy because it is almost universally expressed with high copy numbers on the surface of B-cell lymphomas and is minimally modulated.^17,18^ This study, which followed our prior phase I clinical trial, tested the clinical efficacy of autologous CART-20 cell infusion and evaluated the related toxicities. The results showed that nine of the 11 NHL patients achieved different degrees of clinical remission without serious toxicity. Our results provided further support for the promising applicability of CART-20 in CD20^+^ B-cell malignant diseases, even at an early stage of the diseases.

Based on the anti-tumor activity of the CART-20 cells and the selected delayed toxicities that were reported in seven patients with relapsed or refractory advanced DLBCL in our previous study, we revised the eligibility criteria for patients in this study to optimize the effects of the CART-20 cells. First, an entering criterion for the trial was developed such that maximum diameter ⩾5 cm or number of lesions >3, before infusion, or reduced to be through by chemotherapy, on the basis that an inverse correlation between the degree from clinical benefit of CART-20 and the burden of tumor was observed in our previous study. Although there was an inverse correlation between a detectable persistence of CART cells and the disease burden,^18–20^ large tumor burdens are not totally insensitive and can also be controlled by pretreatment before CART therapy. Second, patients with lung and digestive tract involvement were excluded from this study to avoid massive hemorrhage of the alimentary tract and aggressive intrapulmonary inflammation surrounding extranodal lesions, which were adverse effects observed in the previous clinical trial. Finally, a standard for a unified lymphocyte depletion condition (absolute lymphocytes <0.3×10^9^ l^−1^, regardless of regulatory T or B lymphocytes) was required in every patient before cell infusion to leave more space for proliferation of the CART cells *in vivo*. As expected, all 11 patients achieved varying degree of clinical benefit and 9 of 11 patients achieved remission. Of these, six patients achieved CR. The PFS lasted more than 13 months in four patients (UPN01, -06, -09 and -11), and the longest PFS was up to 27 months at the time of the submission of this manuscript. In the previous study, cytokine release syndrome and tumor lysis symptoms correlated with the tumor burden at the time of the infusion of the CAR-directed T cells.^21,22^ No grade 4 toxicity was found in our phase IIa study in the modified inclusion criteria and debulking treatments were used. This finding indicates that when the CART cells were used in patients earlier in the course of their disease or when pre-infusion conditioning chemotherapy was combined with intensive chemotherapy to reduce the tumor burden, not only was the efficacy of the CART cells improved but the risk of severe cytokine release syndrome was also far lower.

The data from several early clinical trials suggested that high doses of CART cells are associated with increased responses but are accompanied by greater toxicity.^23,24^ Subsequent studies showed that the infused total dose correlated poorly with the steady-state number of cells that engrafted or the persistence of the CART cells. No positive correlation has been found between the T-cell dose and the clinical responses across multiple clinical trials in various settings.^19,20,25–27^ Brentjens *et al.*^18^ reported that T cells could exhibit optimal persistence and antitumor efficacy at a dose of 1×10^7^ 19–28ζ T cells per kg, compared with the initial dose of 3×10^7^ 19–28ζ T cells per kg. We speculated that the CART cells had a high replicative potential and would expand in the host. In our study, we also found that the peak number of circulating CART cells did not correlate with the dose of the infused CART cells or with the percentage of central or effector memory T cells in the T-cell products within the range of 0.41×10^7^–1.46×10^7^ kg^−1^. This difference may be due to the better immune status of patients with lymphoma than patients with leukemia.^28,29^ In addition, the possibility of an error caused by the small sample size should not be ruled out. However, our results suggested the dose of the CART cells in such a range may be sufficient for therapeutic effects without a risk of infusion-related toxicities.

The genetically modified T cells must target the site(s) of malignancy to be able to act on and within the tumor. The results from this study suggest the CART cell responses may not be equivalent at all tumor sites. There has been only one study that reported that the DNA of *CAR* was detected in mouse skin at 10 days after infusion.^30^ Some studies showed CART-19 cells were detected in a skin lymphoma biopsy after CART treatment.^24,31^ In this study, we were the first to use CART-20 to treat a patient with a primary cutaneous marginal zone lymphoma. The skin tissue of this patient achieved CR at 90 days after cell infusion, and the remission has been sustained until our study report submission date. The *CAR* gene was also detected in this patient’s skin biopsy. This patient also had spleen involvement; however, the response of the splenomegaly to the CART-20 treatment was weak. Tabata *et al.*^32^ reported that some indolent splenic lymphoma patients had very low CD20 expression. There have also been reports that some immature cells with a myeloid origin accumulated in the spleen and the tumor bed during the tumor growth and suppressed the anti-tumor immunity in various ways.^33^ These may explain why the spleen did not recruit the CART-20 cells after the first infusion and why the spleen showed a weak response to the CART-20 treatment. However, it was encouraging that the spleen showed continued CR, which was accompanied by the persistence of the *CAR* gene, for more than 9 months after receiving local radiotherapy. A previous study had already reported that various tissue-specific homing receptors, which typically include integrins, chemokines, and chemokine receptors, are associated with the T-cell migration to the anatomic sites of malignancy.^34^ The exposure of antigens on tumor cell surfaces and the microenvironment of the tumor are the major factors that affect the efficacy of CART cell therapy. In some clinical trials, radiotherapy to normalize the often chaotic structure of tumor blood vessels can enhance the effects of the CART cells, and radiation therapy causes tumor necrosis, which releases a large amount of tumor antigens.^35,36^ After receiving radiotherapy, the tumors became necrotic and release large amounts of antigens. The immunosuppressive environment around the tumor is therefore undermined, which is conducive to the effects of the CART on the targets.^37,38^ Hence, new approaches, such as the use of radiation, can be developed to improve the therapeutic efficacy in cases of lymphoma at such resistant sites. Another tumor site with limited response is the testes. Patient UPN02, who had head and neck lymphoma, achieved CR for six months after the cell infusion. However, the tumor recurred at a new site, the testes, after 6 months, and there were few CART-20 cells and a low *CAR* gene copy number in this tumor. It is possible that tumor recurrence after treatment with CART cells occurs at sites that have natural barriers that may exist to protect the site, such as the blood–brain barrier, blood–testis barrier and adipose tissue medium. Some evidence indicates that leukemia cells undergo directed migration and retention within the extramedullary sites of organ infiltration.^39^ Thus, the leukemia cells reside in a microenvironment that facilitates their growth and protects them from spontaneous and induced apoptosis.^40^ Lymphoma cells appear to have the same potential phenomenon of resistance to death, and residual tumor cells transferred to a suitable growth site where the microenvironment could suppress T-cell recruitment would avoid the therapeutic effects of CART.^41^ This mechanism needs further study, and we need to establish new ways to improve the effects of the CART cells in sites where there is insensitivity.

Relapse following CART cell therapy remains a challenge. All of the disease relapses observed in our patients were demonstrated to be CD20-positive. B-cell aplasia continues to result from CART cell persistence. Shannon *et al.*^42^ demonstrated that relapse of acute lymphoblastic leukemia cells that retain surface CD19 expression results from the rapid disappearance of CAR-modified T cells or decreased function of those T cells. In our study, we also observed a positive correlation between the CD20^+^ B-cell aplasia and copies of the genes indicating the CART cells. Therefore, increasing the persistence of the CART cells *in vivo* is an essential prerequisite to avoid tumor relapse. Various measures can prolong T-cell persistence to prevent some of these relapses, including optimization of the CAR designs, manufacturing technologies or T-cell subset ratios. However, repeated infusions appear to be more feasible based on the management of the development of toxicity. Relapse is heralded by recovery of the normal B cells, suggesting that monitoring the return of normal B cells is important to identify patients at the highest risk for relapse, which could potentially provide a window for repeat infusion.

In conclusion, our study confirmed that adoptive immunotherapy with CD20-directed CART cells is a feasible and possibly effective treatment modality for CD20^+^ NHL patients. Debulking regimens combined with a lymphocyte-depleting conditioning strategy prior to CART cell infusions may improve clinical responses and reduce severe toxicity. Finally, combining the CART treatment with local radiotherapy can enhance the therapeutic effects of CART cells at tumor sites, especially in spleens where there the antigen expression is low.

## Figures and Tables

**Table 1:** Patient characteristics and response summary

*Subject number /gender/age*	*Diagnosis/subtypes*	*Stage of disease at first diagnosis*	*IPI/aaIPI*	*The prior therapies*	*Disease status at study entry*	*Conditioning therapy*	*CAR20 kg* ^*−1*^ *(×10* ^*7*^)	*Disease response to CART cells*	*PFS(m)*
UPN01/M/53^a^	DLBCL/ABC	IIISB	2	R-CHOP×5, R-ESHAP×2	SD	None	0.69	CR	13+
UPN02/M/57^b^	DLBCL/ABC	IVA	3	R-CHOP×7	PD	CHOP	0.90	PR	6
UPN03/M/25^b^	DLBCL/ABC	IV/XA	3	CHOP, R-CHOP, R-ICE×3, R-COEP and Auto-HSCT	PD	MACH	0.53	CR	4
UPN04/F/64^a^	DLBCL/ABC	IIA	3	R-CHOP×6	PD	FC	0.41	PR	2
UPN05/M/36^a^	DLBCL/ABC	IVA	4	R-CHOP×6, Radiotherapy and R-GDP×3, GDP, DICE×3	PD	EOCH	0.90	SD	2
UPN06/M/44^a^^,^ c	DLBCL/NOS	IIA	2	CHOP×6, R-CHOP×2, R-ESHAP	SD	CHOD	0.48	PR	(2+11)13+
UPN07/F/46^a^^,^ d	FL	IVA	3	R-CHOP×5, R-FC	PD	None	0.93	CR	5
UPN08/M/49^a^^,^ e	MCL	IIIA	3	R-Hyper-CVAD×5	SD	None	1.01	CR	5
UPN09/M/63^a^^,^ c ^,^ d	PCMZL	IVSA	3	R-CHOP×4, DICE	PD	CHODE	1.46	SD	(3+10)13+
UPN10/M/64^a^	DLBCL/ABC	IIB	3	R-CHOP×2	PR	CHOP	1.29	CR	6+
UPN11/M/70^a^^,^ f	DLBCL/GCB	IIIXB	3	R-CHOP×5, R-ICE×2, R-ESHAP×2 and Radiotherapy	PR	None	0.47	CR	(16+11)27+

Abbreviations: ABC, activated-B-like-cell; CART, Chimeric Antigen Receptor-modified T cells; CR, complete remission; DLBCL, diffuse large B-cell lymphoma; FL, follicular lymphoma; GCB, germinal center B-cell origin; MCL, mantle cell lymphoma; PCMZL, primary cutaneous marginal zone lymphoma; PD, disease progression; PFS, progression-free survival; PR, partial remission; SD, stable disease.

a Refractory.

b Relapsing.

c UPN06 and UPN09 received local radiotherapy at 2 and 3 months after infusion respectively, then they both got complete remission.

d Bone marrow involvement.

e Lost to follow-up because patient refused to come to appointments.

f CART-20 infusion again in phase IIa trail after 16-month CR from our phase I trial.

+ indicates an ongoing response as of the time of manuscript submission.

**Table 2:** Adverse events possibly and probably related to the infusion

*Adverse events*	*AEs* ⩾*grade 3(*n*=11)*	*Any grade(*n*=11)*
ALT elevation	0	2
AST elevation	0	1
Hyperuricaemia	0	2
Hypoalbuminaemia	0	1
Hypokalemia	1	1
LDH elevation	0	2
Exudative inflammation of the lungs	0	1
Herpes zoster	1	1

Abbreviations: ALT, alanine aminotransferase; AST, aspartate aminotransferase; LDH, lactate dehydrogenase.
