# Supplementary material for: Treatment of CD20-directed Chimeric Antigen Receptor-modified T cells in patients with relapsed or refractory B-cell non-Hodgkin lymphoma: an early phase IIa trial report
Source: Signal Transduct Target Ther. 2016 Mar 11;1:16002–. doi: 10.1038/sigtrans.2016.2 (PMC5661644; doi:10.1038/sigtrans.2016.2)
Supplement: Supplementary Information [file sigtrans20162-s1.pdf]

Supplemental Table 1.The dose of CART-20 cells infused to patients

UPN	Total cells ($\times10^9$)	Transfection efficiency%	Total CAR-20 cells ($\times10^7$)	CAR-20/kg ($\times10^7$)
01	1.46	30.51	44.54	0.69
02	1.72	28.09	60.96	0.90
03	0.65	44.63	29.0	0.53
04	0.83	32.17	26.70	0.41
05	1.35	54.13	73.08	0.90
06	1.59	21.29	33.85	0.48
07	1.72	41.00	70.52	0.93
08	3.14	23.42	73.54	1.01
09	3.06	32.82	100.49	1.46
10	1.77	52.44	92.82	1.29
11	1.2	20.36	28.32	0.47

Supplemental Table 2. The ratio of CM and EM cells in CAR positive population.

UPN	CM cells (%)	EM cells (%)
01	4.92	52.58
02	24.72	1.96
03	29.93	34.2
04	0.97	27.36
06	83.72	3.88
10	19.24	31.42

Appendix Figure 1.

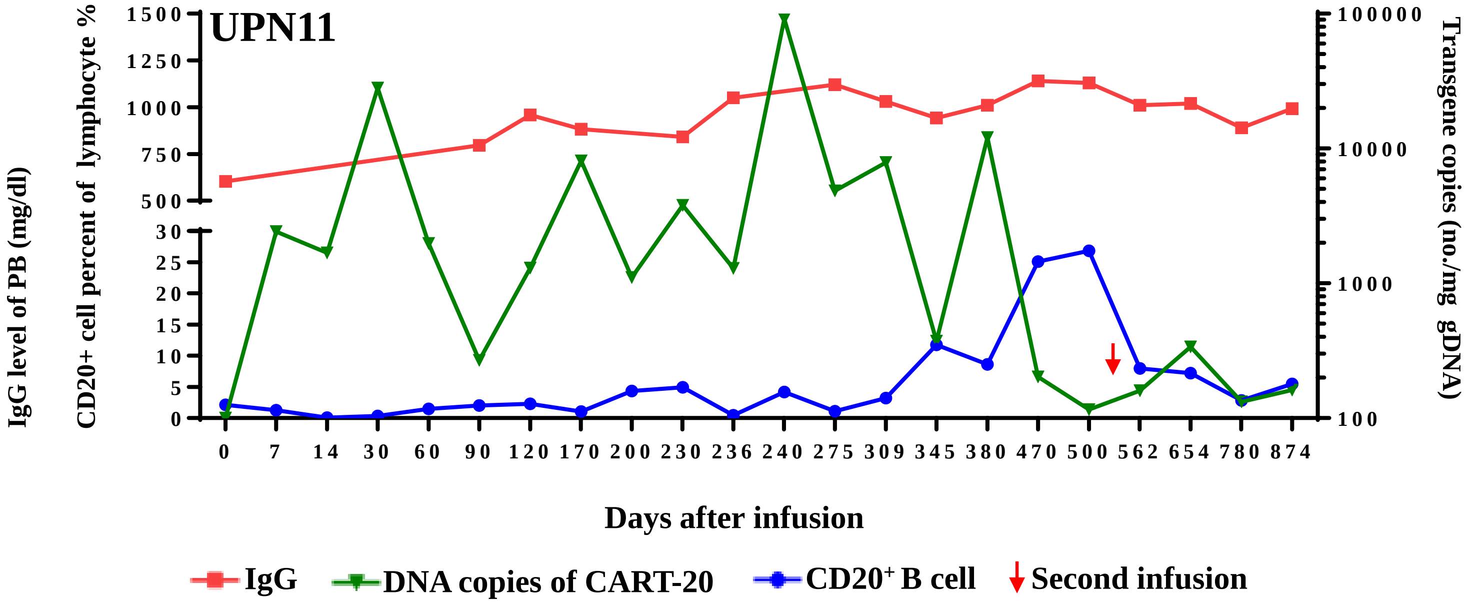

Appendix Figure 1. Time course of the levels of IgG, percentages of CD20+ B cell and DNA copies of CART-20 in the PB of UPN11 following the first infusion. After the first infusion, this patient demonstrated a long-time depletion of CD20+ B cells with fluctuations in the higher levels of DNA copies. However, the IgG levels remained normal because gamma globulin was intermittently replenished. Approximately 500 days after the infusion, the level of CD20+ B cells was increased in parallel with a decrease in the number of copies of the DNA. At this time, a second infusion of CART-20 cells was given, and the increase in the DNA copies and decrease in the CD20+ B cells was observed again.

Appendix Figure 2.

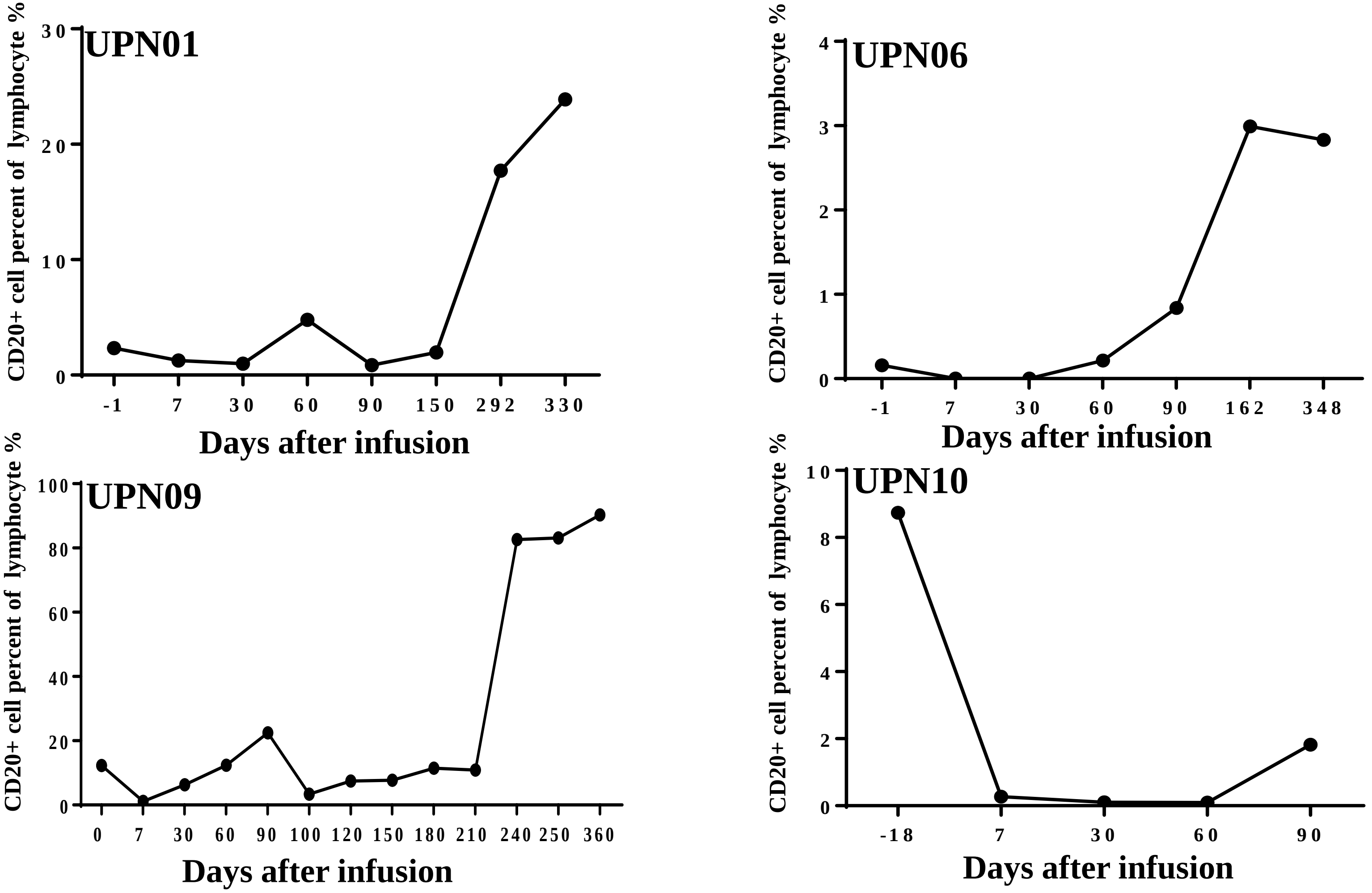

Appendix Figure 2. The CD20+ B cell percentage in lymphocytes of the PB of non-relapsed patients after CART-20 infusion. These four patients achieved sustained remissions but also appeared to show varying degrees of B cell recovery.

Appendix Figure 3.

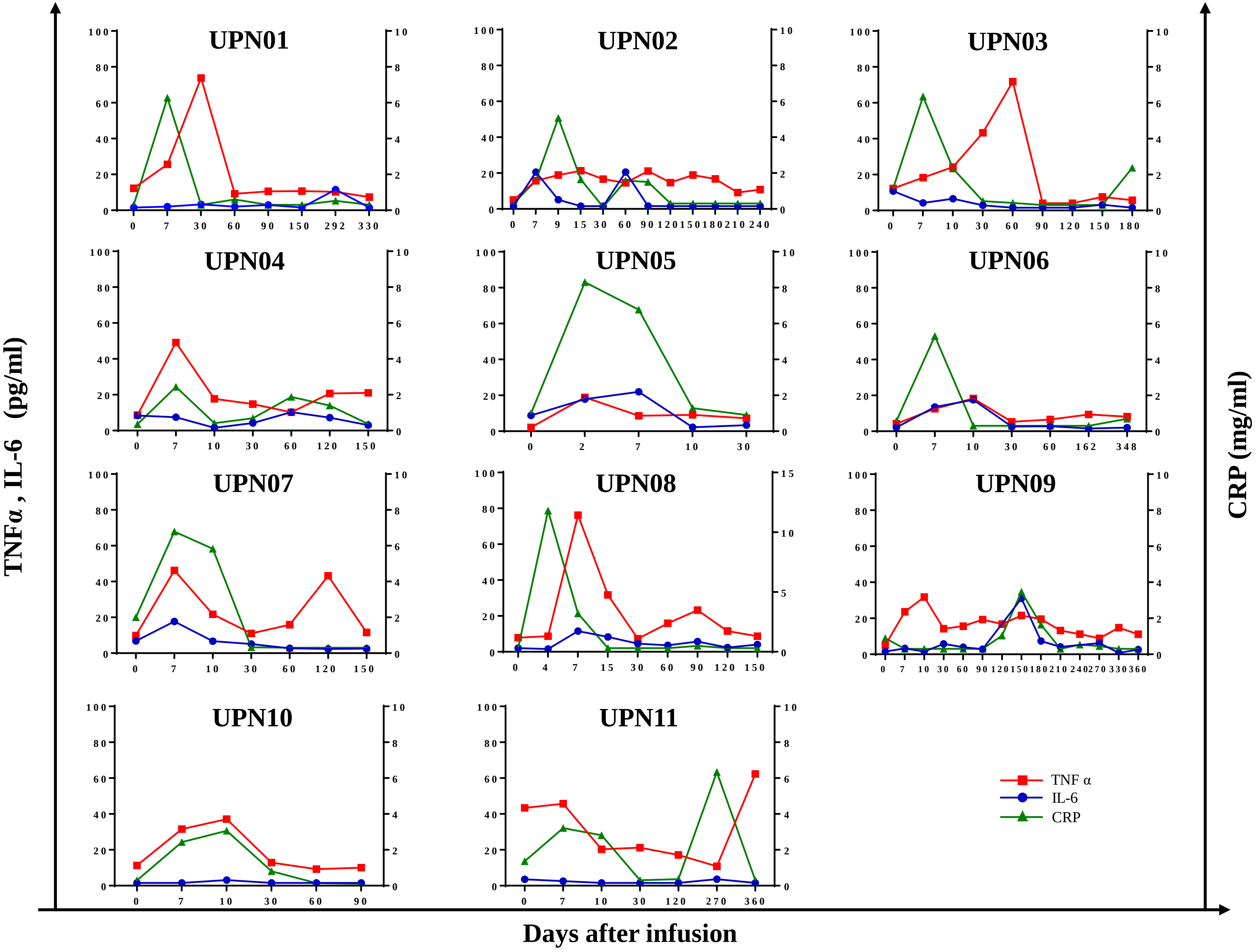

Appendix Figure 3. The levels of TNFα, IL-6 and CRP of PB. The levels of the cytokines begin to rise after the CART-20 infusion, but did not meet the standards for designation as CRS. Eight patients (UPN01, 02, 03, 04, 07, 08, 09 and 11) had secondary waves of increases. Increases in the cytokines of UPN11 were observed after the second infusion.
